# Supplementary material for: A deep neural network model for multi-view human activity recognition
Source: PLoS One. 2022 Jan 7;17(1):e0262181. doi: 10.1371/journal.pone.0262181 (PMC8741063; doi:10.1371/journal.pone.0262181)
Supplement: Attachment — Submitted filename: Response to reviewers.pdf [file pone.0262181.s001.pdf]

Response to Reviewers

Submission ID: PONE-D-20-17086

Response to the comments of reviewers on the paper "A Deep Neural Network Model for Multi-View Human Activity Recognition" reference PONE-D-20-17086.

The authors would like to thank the reviewers for their comments. The responses of the authors to the specific concerns of the reviewers are outlined below.

- **Comment Rev1-1:**

"The use of multiple cameras is a common approach to resolving the issue of occlusion in human activity recognition system."- add reference for this statement. I recommend the following:

- A framework of human detection and action recognition based on uniform segmentation and combination of Euclidean distance and joint entropy-based features selection
- An implementation of optimized framework for action classification using multilayers neural network on selected fused features

Answer Rev1-1:

I am grateful for your comments. I realized that I did not give any references for that statement. Therefore, I added some previous works and your recommendation as references.

**Revision 1-1-(1): Introduction**	
Before	The use of multiple cameras is a common approach to resolving the issue of occlusion in human activity recognition system.
After	The use of multiple cameras is a common approach to resolving the issue of occlusion in human activity recognition systems [1–4].<br><br>1. Sharif M, Khan MA, Akram T, Javed MY, Saba T, Rehman A. A

	framework of human detection and action recognition based on uniform segmentation and combination of Euclidean distance and joint entropy-based features selection.EURASIP Journal on Image and Video Processing. 2017;2017(1):1–18.<br><br>2. Khan MA, Akram T, Sharif M, Javed MY, Muhammad N, Yasmin M. An implementation of optimized framework for action classification using multilayers neural network on selected fused features. Pattern Analysis and Applications.2019;22(4):1377–1397.<br><br>3. Baltieri D, Vezzani R, Cucchiara R. 3dpes: 3d people dataset for surveillance and forensics. In: Proceedings of the 2011 joint ACM workshop on Human gesture and behavior understanding; 2011. p. 59–64.<br><br>4. Torres C, Fried JC, Rose K, Manjunath BS. A multi view multimodal system for monitoring patient sleep. IEEE Transactions on Multimedia.2018;20(11):3057–3068.

- **Comment Rev1-2:**

"Current multi-view approaches involve either conventional computer vision or DNN application."- add the following reference for this statement:

- Human action recognition using fusion of multiview and deep features: an application to video surveillance
- Human action recognition: a framework of statistical weighted segmentation and rank correlation-based selection

Answer Rev1-2:

Thank you for the comments. I added your recommendations as references.

**Revision 1-2-(1): Introduction**	
Before	Current multi-view approaches involve either conventional computer vision or DNN application.

After	Current multi-view approaches comprise conventional computer vision (CV) or DNN applications [7, 8].<br><br>7. Khan MA, Javed K, Khan SA, Saba T, Habib U, Khan JA, et al. Human actioner cognition using fusion of multiview and deep features: an application to video surveillance. Multimedia tools and applications. 2020; p. 1–27.<br><br>8. Sharif M, Khan MA, Zahid F, Shah JH, Akram T. Human action recognition: a framework of statistical weighted segmentation and rank correlation-based selection. Pattern analysis and applications. 2020;23(1):281–294.

- **Comment Rev1-3:**

"The conventional approach requires expert features extraction to identify informative features from raw data, with the features extractor usually working independently from the classifier. Accordingly, studies based on this approach focus either on the classifier or on feature engineering."- add the following references for the validation of this statement:

- A resource conscious human action recognition framework using 26-layered deep convolutional neural network
- A framework of human action recognition using length control features fusion and weighted entropy-variances based feature selection
- Improved strategy for human action recognition; experiencing a cascaded design

Answer Rev1-3:

Thank you for the comments. I added some previous works and your recommendations as references.

**Revision 1-3-(1): Introduction**	
Before	The conventional approach requires expert features extraction to identify informative features from raw data, with the features extractor usually working independently from the classifier. Accordingly, studies based on this approach focus either on the classifier or on feature engineering.

After	The conventional methods require expert features extraction to identify informative features from raw data [9–11]. The features extractor usually works independently from the classifier [12, 13]. Accordingly, studies based on this approach10focus either on the classifier or on feature engineering [14, 15].<br><br>9. Weinland D,˙Ozuysal M, Fua P. Making action recognition robust to occlusions and viewpoint changes. In: European Conference on Computer Vision. Springer;2010. p. 635–648.<br><br>10. Chaaraoui AA, Climent-P´erez P, Fl´orez-Revuelta F. Silhouette-based human action recognition using sequences of key poses. Pattern Recognition Letters.2013;34(15):1799–1807.<br><br>11. K¨ase N, Babaee M, Rigoll G. Multi-view human activity recognition using motion frequency. In: Image Processing (ICIP), 2017 IEEE International Conference on. IEEE; 2017. p. 3963–3967.<br><br>12. Turaga P, Veeraraghavan A, Chellappa R. Statistical analysis on Stiefel and Grassmann manifolds with applications in computer vision. In: Computer Vision and Pattern Recognition, 2008. CVPR 2008. IEEE Conference on. IEEE; 2008. p.1–8.<br><br>13. Spurlock S, Souvenir R. Dynamic view selection for multi-camera action recognition. Machine Vision and Applications. 2016;27(1):53–63.<br><br>14. Khan MA, Zhang YD, Khan SA, Attique M, Rehman A, Seo

	S. A resource conscious human action recognition framework using 26-layered deep convolutional neural network. Multimedia Tools and Applications. 2020; p. 1–23.<br><br>15. Afza F, Khan MA, Sharif M, Kadry S, Manogaran G, Saba T, et al. A framework of human action recognition using length control features fusion and weighted entropy-variances based feature selection. Image and Vision Computing. 2021;106:104090.

- **Comment Rev1-4:**

"Conversely, the DNN approach combines feature extractors and classifiers into a single pipeline. A DNN automatically discovers representation and recognizes the 23 patterns in given data using a general-purpose learning algorithm"- justify this statement.

Answer Rev1-4:

Thank you for the comments. The previous works explained that end-to-end learning algorithms using backpropagation enabled DNN to discover and learn informative representation from the given data for each specific class. Moreover, we presented papers that have applied DNNs to MVHAR.

**Revision 1-4-(1): Introduction**	
Before	Conversely, the DNN approach combines feature extractors and classifiers into a single pipeline. A DNN automatically discovers representation and recognizes the patterns in given data using a general-purpose learning algorithm [9].
After	Conversely, the DNN approach combines feature extractors and classifiers into a single pipeline [19–21]. A DNN model automatically discovers representation and recognizes the patterns in given data using an end-to-end learning algorithm [22]. The algorithm trains the DNN model to extract informative features from the given data for each specific class [23]

	22. LeCun Y, Bengio Y, Hinton G. Deep learning. Nature. 2015;521(7553):436–444.<br><br>23. O'Mahony N, Campbell S, Carvalho A, Harapanahalli S, Hernandez GV,Krpalkova L, et al. Deep learning vs. traditional computer vision. In: Science and Information Conference. Springer; 2019. p. 128–144.

- **Comment Rev1-5:**

Update the related work with more latest articles. I recommend the following:

- Intelligent human action recognition: A framework of optimal features selection based on Euclidean distance and strong correlation
- A Fused Heterogeneous Deep Neural Network and Robust Feature Selection Framework for Human Actions Recognition

Answer Rev1-5:

Thank you for the comments. As the second reviewer also suggested the same thing. We explain the revision in the next section.

- **Comment Rev1-6:**

No need to add the mathematical detail of LSTM. Just add a reference and define the key contributions.

Answer Rev1-6:

Thank you for the comments. I revised the LSTM and Residual learning in LSTM section accordingly.

- Long-short Term Memory (removed)
- Residual learning in LSTM (revised)

**Revision 1-6-(1): Residual learning in LSTM**	
Before	Deeply structured neural networks are widely considered to perform better than the shallower ones, but, deeper neural

	networks yield worse because of degradation problem. Residual learning was proposed to tackle this issue with the introduction of a shortcut
After	A long short-term memory (LSTM) architecture [42] is proposed to solve the problem of vanishing and exploding gradients, associated with conventional recurrent neural networks (RNN) [22]. The architecture, however, still can suffer from degradation problems caused by deeper neural network structure [43]. Residual learning was proposed to tackle this issue with the introduction of a shortcut connection from the earlier to the later layers that helps the earlier layer get a-"fresh"-gradient from the latter one during backpropagation [44].

- **Comment Rev1-7:**

A detailed statistical analysis in term of t-test or any other should be given.

Answer Rev1-7:

I realized that the results lacked statistical analysis. Therefore, I added some statistical analysis to investigate whether a recognition improvement was significant or not. We calculated p-value using Student t test to compare two experimental results.

**Revision 1-7-(1): Related work**
**Addition:**<br>1. Secondly, fine-tuning the last block of pre-trained CNNs improved insignificantly (average of $p > 0.05$) the performance of the proposed model. Accuracy improvements of 4.29% with VGG-16 fine-tuning and 2.52% with VGG-19 fine-tuning were observed.<br>2. An improvement ($p = 0.617$) was observed with the use of shared-weight LSTMRes302in the proposed model (Fig. 5).<br>3. The above exploratory studies indicated that the employment of VGG-16,

shared-weight LSTMRes, and shared fusion with the arithmetic mean significantly improved (p = 0.004) the performance of the proposed model by 7.07±14.03%, compared to the model in our previous work [19].

4. Comparison results of the model using multi-view inputs and single-view inputs demonstrated that combining information from multi-view resulted in significant (average of $p < 0.05$) higher improvement.

- **Comment Rev2-1:**

The English is not perfect. As a sample, consider these sentences in lines 110, 115, 185:

- "Contrary to previous works that attempted to learn weight …"
- "Moreover, this study merely employed raw images as inputs and did not combined …"
- "Previous work by the authors..."

The language has similar problems throughout. Despite the language errors, the actual ideas are generally understandable.

Answer Rev2-1:

Thank you for your comments. We realized that there are some language errors in our manuscript. We proofread our manuscript and revised some sentences, accordingly.

**Revision 2-1-(1): Introduction**	
Before	This paper presents a novel DNN model for multi-view human action recognition based on shared-weight application. Inspired by the ensemble method, the proposed model treated multiple-view images as inputs of multiple models to produce multiple hypotheses. In contrast with previous works [12, 13] that utilized multiple models to process multiple inputs, the proposed model employed a single model with weight-sharing to accomplish that.

	Thereby, our current proposed model had a fewer number of parameters. Employing the Attention layer, features from an input were filtered out to remove irrelevant information; each input was handled by one specific attention layer. Besides, since the prior knowledge about informative inputs among multiple views was unknown, the proposed model was trained to treat the hypotheses of each input equally with arithmetic mean or weight the hypothesis with geometric mean. An attention layer was applied to filter out uninformative features from each input sequence. The model comprises pre-trained CNNs, attention layers, RNN, and softmax layers. Exploration studies were conducted for structural optimization, a transfer learning with pre-trained CNNs was performed to prevent overfitting in the training process. The performance of the proposed model was compared with that of the-state-of-art application on IXMAS [4] and i3DPost [17], and online evaluation was conducted to determine its efficiency in actual situation.
After	This paper presents a novel DNN model using a shared-weight application for multi-view human action recognition. The shared-weight application enabled the model to perform late fusion with fewer parameters than the multi-block technique. The model used multi-view images as inputs to produce multiple hypotheses. Then using the score-fusion, the model computed the final prediction. Since the prior knowledge about informative inputs among multiple views was unknown, we trained the proposed model to treat each prediction score equally with arithmetic mean or weight the hypothesis with geometric mean. The model applied an attention network to filter out uninformative features from each view. The model comprises pre-trained CNNs, attention layers, RNN, and softmax layers. We conducted exploration studies for structural optimization and performed transfer learning with pre-

	trained CNNs to prevent overfitting in the training process. We compared the performance of the proposed model performance to that of the-state-of-art application on IXMAS [17] and i3DPost [27]. We conducted an online evaluation and comparative study with the single-view model to determine its efficiency in the actual situation. We summarize this study's contribution as follow:<br>● We proposed a novel DNN model employing shared-weight for MVHAR. Although the proposed model had fewer parameters, the experimental results demonstrated60that the application of shared-weight yielded higher accuracy than multiple blocks DNN.<br>● We investigated the effect of applying residual shortcuts on LSTM and employing intermediate and last layer of pre-trained CNNs to extract features.<br>● We compared the effectiveness of using features or score fusion to combine information from multi-view inputs.<br>● We evaluated the proposed model on a single-view and multi-view inputs scenario. The experimental results suggested that the multi-view application could increase68the recognition rate.<br>● We demonstrated that the proposed model might be applied in real applications by evaluating it on the online classification scenario.

**Revision 2-1-(2): Related work**	
Before	Contrary to previous works that attempted to learn weight or adjust adaptively the weight for each input [20], the proposed model assumed that prior knowledge of informative view was

	unknown, thereby the model treated each hypothesis from view-input equally with arithmetic mean or weighted it with geometric mean during the training and did not need to adjust the weight for each input during the inference.
After	Previous works combine the scores with weighting functions whose parameters' values were determined during training [8] or inference [36]. In contrast, the proposed model assumed that prior knowledge of informative views was unknown. Therefore, the model treated prediction score from each view input equally with arithmetic mean or weighted it with geometric mean during training and inference.

**Revision 2-1-(3): Related work**	
Before	Moreover, this study merely employed raw images as inputs and did not combined the latent variables with another modality.
After	The proposed model employed RGB images as inputs and did not combine the latent variables with another modality.

**Revision 2-1-(4): Shared weight LSTMRes**	
Before	Previous work by the authors employed Multiple Sequence LSTMRes (MSLSTMRes) to decode temporal deformation changes in features from multiple cameras [12].
After	The model in our previous work used Multiple Sequence LSTMRes (MSLSTMRes) to decode temporal deformation changes in features from multiple cameras [19].

**Revision 2-1-(5): Exploration studies**	
Before	This section details the results of exploration studies using the IXMAS dataset, with evaluation of LSTMRes performance in comparison to LSTMResKim, LSTM, and Convolutional LSTM

	(ConvLSTM) [29]. The impacts of fine-tuning pre-trained CNNs were compared to those of the proposed model. VGG-19 and VGG-16 were used in this experiment after research with other models such as ResNet [25] and Inception [30], which unexpectedly impaired the recognition rate of the proposed model. The performance of a multiple stacking approach and shared-weight topology was also evaluated, and the effects of score fusion in the model were analyzed. The experimental protocols used in the experiments varied (Section Pre-processing and learning), and in every experiment, the most optimal structure for the succeeding experiment was identified.
After	This section details the results of exploration studies using the IXMAS dataset. The experiments included:<br>1. a performance comparison of LSTMRes with LSTMResKim [46], LSTM, and Convolutional LSTM (ConvLSTM) [49],<br>2. an investigation on the impacts of fine-tuning pre-trained CNNs with VGG-19 and VGG-16; other models such as ResNet [44] and Inception [50] were not used because they impaired the recognition rate of the proposed model,<br>3. an evaluation on the performance of a multiple-block approach and shared-weight topology, and<br>4. a comparison of features fusion with score fusion using arithmetic and geometric means.<br>The experimental protocols used in the experiments varied (Section Pre-processing and learning). And in every experiment, the most optimal structure was used for the succeeding experiment.

- **Comment Rev2-2:**

The literature review is missing some relevant papers such as:

"Dividing and Aggregating Network for Multi-view Action Recognition" by Wang et al.
"Cross-View Action Recognition via Transferable Dictionary Learning" by Zheng et al.
Many others can be found in their related works sections.
Some of the suggested approaches perform experiments on the same datasets taken in consideration by the authors. Others perform experiments on different datasets but this should not be a valid reason to not mention them.

Answer Rev2-2:

I realized that there are some relevant papers were not included in the related work section. Therefore, I added some previous works and your recommendations in that section.

**Revision 2-2-(1): Related work**	
Before	In the last decade, multi-view action recognition has been dominated by conventional CV approaches that represent human body configuration using 2D, 3D, or 4D models. Methods utilizing 2D models typically involve advance extraction of silhouettes and optical flow from sequences of images for direct classification and transformation [2] to higher level features [8,18,19]. Silhouettes contour points and centers of mass have been identified as informative features for movement in human action [2], and, high-level feature representation has demonstrated superiority over other methods involving the employment of 2D data [8]
After	In the last decade, conventional CV approaches have dominated the MVHAR field; they represented human body configuration using 2D, 3D, or 4D models. Methods utilizing 2D models extracted silhouettes and optical flow from sequences of images for direct classification [10, 16] or transformation to higher-level features [13, 32–35]. High-level features such as silhouettes contour points and centers of mass [10] have demonstrated superiority over other methods involving the employment of 2D data [13] to encoded movement in human action.

Revision 2-2-(2): Related work	
Before	As examples of learning representation with CNNs over expert-designed features, multi-view methods based on DNNs have been proposed in recent works [12{14,16,21]. Kavi et al. [13] proposed a DNN model that comprised multiple blocks to handle multiple inputs; score fusion from all models was performed to determine human action from multiple views. Despite its promising performance, multiple block DNNs models required a large number of parameters to compromise the number of inputs. To tackle that issue, Khan et.al [14] employed a single pre-trained model to extract features from multi-view inputs and combined them with hand-crafted features. This technique allowed the proposed model to estimate human action from multi-view with fewer parameters. To improve classification performance, recent studies leveraged information from multiple by adding more features to the model such as optical flow [16] or training it to explore latent connections in cross-view aspects [21]
After	As examples of learning representation with CNNs, multi-view methods based on DNNs also have been proposed in recent works [19, 21, 26, 28, 30, 37]. Kavi et al. [26] proposed a DNN model that comprised multiple blocks to handle multi-view inputs; they combined prediction scores from all models to determine human action. Besides, the multi-branch of CNN has been used to perform view-specific action recognition from multiple views using view-specific classifier [21, 37]. Despite their promising performance, multiple block DNNs models required a significant number of parameters to compromise the number of inputs. To tackle that issue, Khan et. al [28] employed a single pre-trained model to extract features from multi-view inputs and combined

	them with hand-crafted features. This technique allowed the proposed model to estimate human action from multi-view with fewer parameters. Recent studies have attempted to improve further the DNN's recognition rate in MVHAR by training the model with multiple modalities [30], or multi-task and cross-view [37] techniques. Multimodal approaches combined 2D-RGB images with higher-level inputs such as optical flow [30, 36] and skeleton features [38]. Multi-task assumed that the model could produce informative latent variables by simultaneously learning different but related tasks: predicting human activities from multi-view inputs and the inputs' view-index [20]. Cross-view methods trained the model to predict how the given activities videos were seen from a different viewpoint [31, 37]; the methods used inputs from different views during training and test.<br><br>20. Vyas S, Rawat YS, Shah M. Multi-view action recognition using cross-view video prediction. In: Computer Vision–ECCV 2020: 16th European Conference, Glasgow, UK, August 23–28, 2020, Proceedings, Part XXVII 16. Springer; 2020.p. 427–444.<br><br>21. Wang D, Ouyang W, Li W, Xu D. Dividing and aggregating network for multi-view action recognition. In: Proceedings of the European Conference on Computer Vision (ECCV); 2018. p. 451–467.<br><br>26. Kavi R, Kulathumani V, Rohit F, Kecojevic V. Multiview fusion for activity recognition using deep neural networks. Journal of Electronic Imaging.2016;25(4):043010–043010.<br><br>28. Khan MA, Sharif M, Akram T, Raza M, Saba T, Rehman A. Hand-crafted and deep convolutional neural network features

	fusion and selection strategy: an application to intelligent human action recognition. Applied Soft Computing.2020;87:105986<br>31. Zheng J, Jiang Z, Chellappa R. Cross-view action recognition via transferable dictionary learning. IEEE Transactions on Image Processing.2016;25(6):2542–2556.<br><br>36. Gao Z, Xuan HZ, Zhang H, Wan S, Choo KKR. Adaptive fusion and category-level dictionary learning model for multiview human action recognition. IEEE Internet of Things Journal. 2019;6(6):9280–9293.<br><br>37. Wang L, Ding Z, Tao Z, Liu Y, Fu Y. Generative multi-view human action recognition. In: Proceedings of the IEEE/CVF International Conference on Computer Vision; 2019. p. 6212–6221.

- **Comment Rev2-3:**

In the reviewer opinion, the experiments section should compare the authors framework for multi-view activity recognition with standard methods for single-view activity recognition. It is not always true that more than one viewpoint reduces errors in the recognition. In general, adding irrelevant information can lead to errors and this should be taken in consideration by the authors. It should be demonstrated that their framework is able to tackle irrelevant information and not just stated as in lines 49-51.

Answer Rev2-3:

I agreed with the reviewer's comment. Hence, I performed experiment using the proposed model with single-view input. Comparison results of the model's performance with single-view and multi-view inputs were presented in "Comparison with a single-input model" section.

**Revision 2-3-(1): Comparison with a single-input model**
**Addition:**<br>An experiment on IXMAS with 13 actions was performed to investigate the proposed

model's performance using multi-view and single-view inputs. Comparison results (Fig. 8) of the model using multi-view inputs and single-view inputs demonstrated that combining information from multi-view resulted in significant (average of $p < 0.05$) higher improvement. Also, the results signified that utilizing inputs from Cam4 caused the model to produce a 37.18% lower recognition rate while using information from the other views yielded a 15.92±1.23% lower accuracy rate.

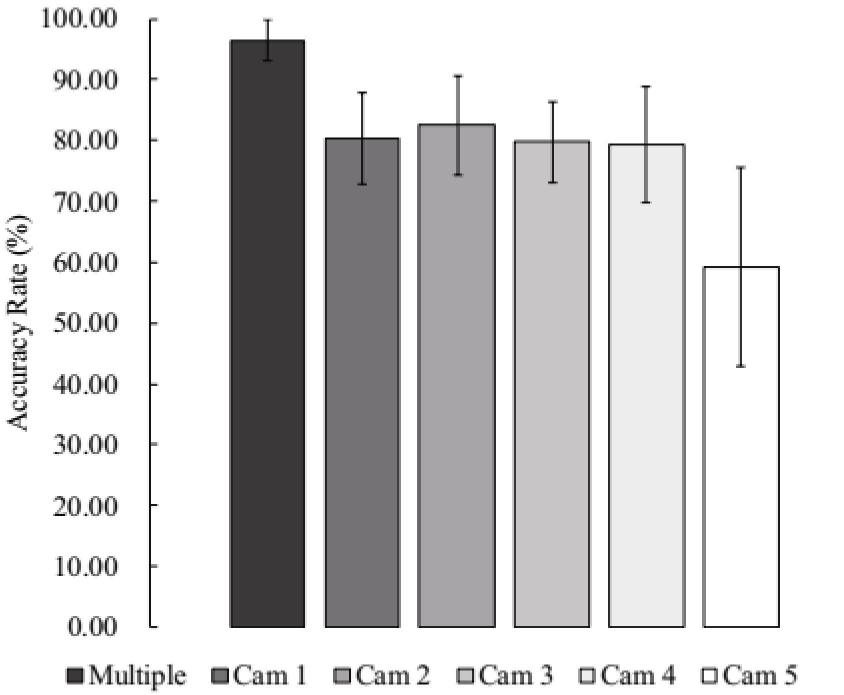

Fig 8. Recognition rate of proposed model utilizing multiple-view inputs (93.67 ± 3.39%) and single-view inputs from Cam 1 (80.34 ± 7.57%), Cam 2 (82.487 ± 8.10%), Cam 3 (79.70 ± 6.66%), Cam 4 (79.27 ± 9.56%), and Cam 5 (59.19 ± 16.37%).

Revision 2-3-(2): Discussion

Addition:

Similar to previous works' finding [34, 35], this study found that combining information from multiple views resulted in a higher accuracy rate of the proposed model in MVHAR. That indicated that information loss caused by occlusion might be compromised with additional information from another view. Also, the results suggested that the proposed model could filter out uninformative features since its

recognition rate did not decline when combining input from Cam4 with the others; the use of single-view input from Cam4 impaired the proposed model's performance.<br><br>34. Liu AA, Su YT, Jia PP, Gao Z, Hao T, Yang ZX. Multiple/single-view human action recognition via part-induced multitask structural learning. IEEE transactions on cybernetics. 2014;45(6):1194–1208.<br><br>35. Liu AA, Xu N, Su YT, Lin H, Hao T, Yang ZX. Single/multi-view human action recognition via regularized multi-task learning. Neurocomputing.2015;151:544–553.

- **Comment Rev2-4:**

In the reviewer opinion, the framework code should be provided.

Answer Rev2-4:

Source code: https://github.com/gitarja/HumanActivityRecognition

- **Comment Rev2-5:**

Why any network can be used for the attention network? It should be demonstrated

Answer Rev2-5:

Previous work has demonstrated that dense, CNN, and RNN layers can be used to produce attention probability. In our preliminary experiments, CNN and RNN impaired the proposed model's accuracy rate; overfitting occurred when utilizing those layers. Therefore, we decided to use dense layer.

**Revision 2-5-(1): Attention network**	
Before	Any network can be used for the attention network; in the proposed model feed-forward neural network is utilized.
After	Dense, convolutional, and RNN layers can be utilized as attention network [40]; the proposed model employed a dense layer for the attention network because, in the preliminary experiment, we found CNN and RNN caused overfitting.<br><br>40. Vaswani A, Shazeer N, Parmar N, Uszkoreit J, Jones L,

	Gomez AN, et al. Attention is all you need. In: Advances in neural information processing systems;2017. p. 5998–6008.

- **Comment Rev2-6:**

Why the relevant features are determined with Hadamart product? There should be an explanation for using such a formula.

Answer Rev2-6:

I added explanation for that formula.

**Revision 2-6-(1): Discussion**
**Addition:**<br>Eq. 4 weights features extracted from pre-trained CNNs with αt.

- **Comment Rev2-7:**

It is not the case to discredit the work done in [35]. In [35] the experiments are realized on Weizmann and Kth datasets. In order to compare the authors framework with [35], the experiments should be conducted on the same datasets. In general, the number of frames to effectively detect an activity really depends on the activity itself.

Answer Rev2-7:

I agreed with your comment since our work and work done in [35] utilized different datasets. Therefore, I revised the statement, accordingly.

**Revision 2-7-(1): Discussion**	
Before	Implementation of the proposed model in an online scenario suggested that a longer sliding window was required for optimal performance. This contradicts the outcomes of previous work [35] stating that a short sequence is sufficient for evaluation of basic human actions. In the experimentation reported here, short sequences resulted in ambiguous clips, thereby impairing the

	performance of the proposed model.
After	Implementation of it in an online scenario indicated that the proposed model required a longer sliding window to attain optimal performance. That contradicts the results of previous work [55] that found a short sequence was sufficient for the evaluation of basic human actions. One interpretation of these findings is that the appropriate number of frames to recognize human activities may differ from case to case; we utilized different datasets from Schindler et al. [55]. In our experiment, short sequences resulted in ambiguous clips, thereby impairing the performance of the proposed model.
