# Supplementary material for: A deep neural network model for multi-view human activity recognition
Source: PLoS One. 2022 Jan 7;17(1):e0262181. doi: 10.1371/journal.pone.0262181 (PMC8741063; doi:10.1371/journal.pone.0262181)
Supplement: Attachment — Submitted filename: Response to Reviewers.pdf [file pone.0262181.s002.pdf]

Response to Reviewers

Submission ID: PONE-D-21-30206

Response to the comments of reviewers on the paper "A Deep Neural Network Model for Multi-View Human Activity Recognition" reference" PONE-D-21-30206.

We would like to thank the reviewers for their comments. We write the responses to the reviewers' specific concerns in the following sections:

Comments from Reviewer 1:

- **Comment 1**

In the introduction section, it is essential to discuss the applications of single view and multi-view in the revised version.

Response:

We are grateful for your comments. We realized we did not explain the applications of single-view and multiple-view. However, as the focus of our study is to estimate human activity, we add explanation about limitation of the single-view human activity recognition and how previous studies tried to solve that problem by employing multiple cameras to compromise complete loss information that can happen in single-view method.

**Revision 1-1: Introduction**	
Before	The use of multiple cameras is a common approach to resolving the issue of occlusion in human activity recognition system. This method allows the captures of human actions from different viewpoints, thereby enabling 3D posture representation. Multi-view action recognition has broad applications ranging from surveillance to classification of human behavioral systems.
After	Occlusion causes information loss and failure in single-view human activity recognition [1]. Previous researchers [2–7] have attempted to resolve this issue with the use of multiple cameras providing different angles of view [1,6] and enabling 3D posture representation [8,9]. Multi-view human action

	recognition (MVHAR) has a wide range of applications, including in systems for surveillance [4,10] and human behavior monitoring [5,11].

- **Comment 2**

Discuss the purpose of multi-view and also describe its importance with related references. it is missing in the revised version.

Response:

In the revised version, we added explanation of how previous researches attempted to solve information loss when self or inter-object occlusion occurred in single-view technique by using multiple-view method. Multiple-view method could compromise the information loss by providing information from other cameras. Our findings agreed with the previous results' findings: employing multiple-view could improve the performance of the proposed model. We discussed this point in Discussion section.

**Addition 1-2: Discussion**	
	As seen in previous works [1,6], this study found that combining information from multiple views resulted in a higher accuracy rate of the proposed model in MVHAR. This suggested that additional information from another view mitigated information loss caused by occlusion. The results also suggested that the proposed model could filter out uninformative features, since the recognition rate did not decline when input from Cam5 was combined with other views; using single-view input from Cam5 resulted in impaired performance of the proposed model.

- **Comment 3**

The problem statement should be modified like what are the problems in single view and what is the need of multi-view?

Response:

Thank you for this comment. We added this point to the Introduction section. We highlighted issue of employing only single-view camera and how the use of multiple-view can solve that problem.

- **Comment 4**

The last contribution is just an experiment?

Response:

We realized we had not explained the detail of the last contribution. We conducted an experiment in an online scenario to investigate the performance of the proposed model to estimate ambiguous actions in online classification and to understand the length of clip needed to get satisfying performance. We found the model could achieve competitive performance in online classification. The results related to the length of the clip, however, disagreed with the previous results: previous results found that shorter sequence resulted in higher recognition rate. The discrepancy might be caused by the different dataset in our and their experiments. In brief, the length of clip needed to get satisfying performance may differ in one case to another.

**Addition 1-4: Introduction**	
	Evaluation of the model's performance during actual application in online classification indicated that longer image sequences produced higher recognition rates.
**Addition 1-4: Discussion**	
	Another major finding of this study was that the proposed model required a longer sliding window to attain optimal performance. That contradicts the results of previous work [65] that found a brief sequence was sufficient for the evaluation of basic human actions. One interpretation of these findings is that the applicable number of frames to recognize human activities may differ from case to case; we used different datasets from Schindler et al. [65]. In our experiment, short sequences resulted in ambiguous clips, impairing the performance of the proposed model.

- **Comment 5**

What are the parameters of LSTM? Have you make any changes?

Response:

We added the detail of how we initialized the LSTM and LSTMRes layers to Preprocessing and Learning section.

**Addition 1-5: Preprocessing and learning**	
	Glorot uniform [57] and orthogonal [58] initializers were used to initialize the parameter values of kernel and recurrent weights, respectively; the bias values were initialized to be zero. As this study used pre-trained CNNs in all experiments, it did not apply parameters initialization to CNNs.

- **Comment 6**

What are the parameters of CNN? which layer is selected for feature mapping?

Response:

We used the parameter value of pre-trained CNNs in our experiment. We did not initialize the parameter values by ourselves. Regarding the layer for feature mapping, we explained it in the Exploration studies section. Considering the results of the exploration study, we used block5_pool of VGG-16 as the layer for feature mapping in our proposed model.

- **Comment 7**

I am worried that why you select only two datasets? why not select UCF101 and HMDB51? Add this discussion in the future work.

Response:

Thank you for your suggestion. We designed the proposed model to estimate human activity from synchronized multiple cameras. Hence, we used IXMAS and i3DPost benchmark datasets to evaluate the model; UCF101 and HMDB51 consist video clips of complex human activity from a single view camera. However, we believe we need to evaluate the proposed model with more complex human activity dataset taken with unsynchronized multiple cameras. We add this point as the future works.

**Addition 1-7: Discussion**	
	Despite promising results achieved with the proposed model compared to state-of-the-art methods, this study had several limitations. First, the model did not get satisfying results evaluated for online scenario requiring

	classification of sequences of ambiguous action. Second, the study only evaluated the proposed model's performance with two benchmark datasets, which comprised less than 15 subjects performing basic activities. Hence, the experimental results remain preliminary. Last, only self-occlusion was observed in the datasets. Accordingly, the proposed model's performance for mutual occlusion is unclear. Further study is needed to evaluate and improve model performance in an online scenario involving more subjects. It also would be of interest to consider evaluating the model with more benchmark datasets comprising complex activities performed in various situations, such as CASIA [66], UCF101 [67], MOD20 [68], and HMDB51 [69] datasets.

- **Comment 8**

The related work should be refine further and add the latest articles.. The following articles can be added.

- A Fused Heterogeneous Deep Neural Network and Robust Feature Selection Framework for Human Actions Recognition

- Real-time Violent Action Recognition Using Key Frames Extraction and Deep Learning

- Multi-Layered Deep Learning Features Fusion for Human Action Recognition

- Video Analytics Framework for Human Action Recognition

Response:

We are grateful for your comments. We refined the related works with more recent works. Most of the new related works are DNN based methods that combine DNN features with hand-crafted features, use multimodal inputs, and employ multi-task technique.

New references:

1. Khan, M. A., Zhang, Y. D., Alhusseni, M., Kadry, S., Wang, S. H., Saba, T., & Iqbal, T. (2021). A fused heterogeneous deep neural network and robust feature selection framework for human actions recognition. Arabian Journal for Science and Engineering, 1-16.
2. Xia, L. M., Guo, W. T., & Wang, H. (2020). Interaction behavior recognition from multiple views. Journal of Central South University, 27(1), 101-113.
3. Li, T., Fong, S., Wong, K. K., Wu, Y., Yang, X. S., & Li, X. (2020). Fusing wearable and remote sensing data streams by fast incremental learning with swarm decision table for human activity recognition. Information Fusion, 60, 41-64.
4. Lekic, V., & Babic, Z. (2019). Automotive radar and camera fusion using Generative Adversarial Networks. Computer Vision and Image Understanding, 184, 1-8.
5. Gu, Y., Ye, X., Sheng, W., Ou, Y., & Li, Y. (2020). Multiple stream deep learning model for human action recognition. Image and Vision Computing, 93, 103818.
6. Hwang, H., Jang, C., Park, G., Cho, J., & Kim, I. J. (2020). Eldersim: A synthetic data generation platform for human action recognition in eldercare applications. arXiv preprint arXiv:2010.14742.

Comments from Reviewer 2:

All the comments to the authors were satisfactorily addressed. The related work section was improved, in the experiments section the authors model is now evaluated in a single-view scenario. The English is still not perfect.

Response:

We are grateful for your comments. We send our revised manuscript to a proofreader and changed it according to feedbacks from the proofreader. We write some samples of the revisions in this document. For all revisions, please check track changes file to see details of the revised parts.

**Revision 2-1: Abstract**

Before	In multi-view human activity recognition, multiple cameras are used to resolve issues relating to occlusion. In this context, previous studies of multi-view methods have been dominated by conventional computer vision approaches that require expert-designed feature extractors. With the success of learning representation using deep neural networks (DNNs), recent works have proposed DNNs models to estimate human activity from multi-view inputs. But, a small number of datasets makes it difficult to train the models to have promising generalization performance. Therefore, this paper presents a DNN model, trained with transfer learning, to classify human action with multiple cameras. The model comprises pre-trained convolutional neural networks (CNNs), attention layers, long-short-term memory with residual learning (LSTMRes), and softmax layers. Utilizing shared-weight allows the proposed model to perform late fusion using a single model. The proposed model achieved a promising performance with challenging multi-view action recognition datasets: IXMAS (97.27%) and i3DPost (96.87%). Also, the evaluation of it on online evaluation demonstrated the proposed model's real-time performance.
After	Multiple cameras are used to resolve occlusion problem that often occur in single-view human activity recognition. Based on the success of learning representation with deep neural networks (DNNs), recent works have proposed DNNs models to estimate human activity from multi-view inputs. However, currently available datasets are inadequate in training DNNs model to obtain high accuracy rate. Against such an issue, this study presents a DNNs model, trained by employing transfer learning and shared-weight techniques, to classify human activity from multiple cameras. The model comprised pre-trained convolutional neural networks (CNNs), attention layers, long short-term memory networks with residual learning (LSTMRes), and SoftMax layers. The experimental results suggested that the proposed model could achieve a promising performance on challenging MVHAR datasets: IXMAS (97.27%) and i3DPost (96.87%). A competitive recognition rate was also observed in online classification.

Revision 2-2: Introduction	
Before	The use of multiple cameras is a common approach to resolving the issue of occlusion in human activity recognition systems [1–4]. This method captures human actions from different viewpoints, thereby enabling 3D posture representation. Multi-view human action recognition (MVHAR) has broad applications ranging from surveillance [3,5] to human's behavior monitoring [4,6] systems.
After	Occlusion causes information loss and failure in single-view human activity recognition [1]. Previous researchers [2–7] have attempted to resolve this issue with the use of multiple cameras providing different angles of view [1,6] and enabling 3D posture representation [8,9]. Multi-view human action recognition (MVHAR) has a wide range of applications, including in systems for surveillance [4,10] and human behavior monitoring [5,11].

Revision 2-3: Introduction	
Before	Conversely, the DNN approach combines feature extractors and classifiers into a single pipeline [22–24]. A DNN model automatically discovers representation and recognizes the patterns in given data using an end-to-end learning algorithm [25]. The algorithm trains the DNN model to extract informative features from the data for each specific class [26]. DNNs have outperformed the conventional methods in many fields of the CV, such as object recognition [27] and video classification [28]. Yet, to the authors' knowledge, performances of current methods using DNNs have not matched that of state-of-the-art methods [22,29]. One reason is the difficulty of training DNN with the limited number of samples in MVHAR datasets [21,30].
After	Conversely, the DNN approach combines feature extractors and classifiers into a single pipeline [19–21]. A DNN model automatically discovers representation and recognizes the patterns in given data using an end-to-end learning algorithm [22]. The algorithm trains the DNN model to extract

	informative features from the given data for each specific class [23]. DNNs have outperformed the conventional methods in many fields of the CV, such as object recognition [24] and video classification [25]. Yet, to the authors' knowledge, the performance of current methods utilizing DNNs has not matched that of state-of-the-art applications [19,26]. This was attributed to the difficulty of training DNN with the limited number of samples in MVHAR datasets [17,27].

**Revision 2-4: Introduction**	
Before	This paper presents a novel DNN model using a shared-weight application for multi-view human action recognition. The shared-weight application enabled the model to perform late fusion with fewer parameters than the multi-block technique. The model used multi-view images as inputs to produce multiple hypotheses. Then using the score-fusion, the model computed the final prediction. Since the prior knowledge about informative inputs among multiple views was unknown, we trained the proposed model to treat each prediction score equally with arithmetic mean or weight the hypothesis with geometric mean. The model applied an attention network to filter out uninformative features from each view.<br>The model comprises pre-trained CNNs, attention layers, RNN, and softmax layers. We conducted exploration studies for structural optimization and performed transfer learning with pre-trained CNNs to prevent overfitting in the training process. We compared the performance of the proposed model performance to that of the-state-of-art application on IXMAS [17] and i3DPost [27]. We conducted an online evaluation and comparative study with the single-view model to determine its efficiency in the actual situation.
After	This paper presents a novel DNNs model based on a shared-weight application for multi-view human action recognition, supporting late fusion with fewer parameters than the multi-model technique. The model involved the use of multi-view images as input to produce multiple hypotheses, and score-fusion was used to compute the final prediction. As the prior details of

	informative input among multiple views were unknown, the proposed model was trained to treat individual prediction scores equally with the arithmetic mean or weight the hypothesis with the geometric mean. The model applied an attention network to filter out uninformative features from the sequence of images.<br>The model comprises pre-trained CNNs, attention layers, RNNs, and Softmax layers. Exploration studies were conducted for structural optimization, and transfer learning was performed with pre-trained CNNs to prevent over-fitting in the training process. We compared the performance of the proposed model performance to that of the-state-of-art application on IXMAS [22] and i3DPost [31]. We conducted an online evaluation and comparative study with the single-view model to determine its efficiency in the actual situation.

**Revision 2-5: Related work**	
Before	In contrast with features-fusion methods, score-fusion approaches treated features from each input individually and then combined the hypotheses from all inputs, using the weighting functions. Previous works have been fusing the scores using several techniques, for example, arithmetic mean [26], fixed weight operation [8], and data-driven adaptive weight algorithm [35]. Arithmetic means assumed that prior knowledge of informative views is unknown; thereby, all confidence scores were treated equally [26]. Fixed weight operation learned to find which views were more informative from the given data [8], while adaptive weight did not put any assumption and produced different weights for different inputs during the inference [35].
After	In contrast with features-fusion, score-fusion involves separate treatment of input features, followed by a combination of hypotheses from all inputs using weighting functions. Previous works have involved score-fusion using the arithmetic mean [30], fixed weight operation [13], and a data-driven adaptive weight algorithm [21]. Arithmetic means assumed that prior knowledge of informative views was unknown; it treated all confidence scores equally [30].

	Fixed weight operation involved learning to identify informative inputs from the data [13], while adaptive algorithm weighted the hypothesis with different masks during the inference [21].

**Revision 2-6: Shared weight LSTMRes**	
Before	Here, this issue was addressed using shared weights of pre-trained CNNs and stacked LSTMRes across inputs from all cameras. The stacked LSTMRes comprises two LSTMRes with 512 units. In previous work, a shared hidden-layer network has been applied to find similarities in speech and text [46]. This work employed a shared-weight model to learn transformation and similarity among features from multiple cameras. And it allows the model to perform late fusion using only a single model.
After	To address that issue, the proposed model used shared weights of pre-trained CNNs and stacked LSTMRes (comprising two LSTMRes with 512 units) across inputs from all cameras. Previous work applied a shared hidden-layer network to find similarities in speech and text [55]. This work employed a shared-weight model to learn transformation and similarity among features from multiple cameras, enabling late fusion using only a single model.

**Revision 2-7: Comparison with a single-input model**	
Before	An experiment on IXMAS with 13 actions was performed to investigate the proposed model's performance using multi-view and single-view inputs. Comparison results (Fig. 10) of the model using multi-view inputs and single-view inputs demonstrated that combining information from multi-view resulted in significant (average of $p < 0.05$) higher improvement. Also, the results signified that utilizing inputs from Cam4 caused the model to produce a 37.18% lower recognition rate while using information from the other views yielded a 15.92 ± 1.23% lower accuracy rate.
After	We performed an experiment on IXMAS with 13 actions to evaluate the proposed model's performance using single-view and multi-view inputs.

	Comparison of results (Fig 10) demonstrated that combining information from multi-view inputs produced a significant improvement in accuracy rate by 20.17 ± 8.57% (n = 396, average $p < 0.05$). Compared to multi-view applications, the outcomes also indicated that the use of input from Cam5 (top-view) produced a 37.18% lower recognition rate, while the employment of the other views yielded a 15.92 ± 1.23% lower accuracy rate.

**Revision 2-8: Comparison with state-of-the-art methods**	
Before	In evaluation with IXMAS, the proposed model significantly outperformed all 2D methods in recognizing 11 actions by 12.05% on average (Table 3). Performance was higher than with 3D methods [17,18] but was slightly lower than the outcomes reported in [12]. Also, the proposed model obtained a 4.46% higher accuracy rate than other DNN models using 2D inputs and achieved comparable results to the models utilizing 2D + optical flow inputs. Yet, the proposed model's accuracy rate was 2.33% lower than the method's employing adaptive score fusion.
After	In evaluation with IXMAS, the proposed model outperformed all 2D methods in recognizing 11 actions by 12.05% on average (Table 3). Performance was higher than methods employing 3D features representation [8,22] but was slightly lower than the outcomes reported in [17]. The proposed model also got a 4.46% higher accuracy rate than other DNN models using 2D inputs and achieved competitive results to the models using 2D + optical flow inputs. However, the accuracy rate of the proposed model was 2.33% lower than that of the adaptive score fusion method.

**Revision 2-9: Discussion**	
Before	Similar to previous works' finding [?,34], this study found that combining information from multiple views resulted in a higher accuracy rate of the proposed model in MVHAR. That indicated that information loss caused by occlusion might be compromised with additional information from another view. Also, the results suggested that the proposed model could filter out

	uninformative features since its recognition rate did not decline when combining input from Cam4 with the others; the use of single-view input from Cam4 impaired the proposed model's performance.
After	As seen in previous works [1,6], this study found that combining information from multiple views resulted in a higher accuracy rate of the proposed model in MVHAR. This suggested that additional information from another view mitigated information loss caused by occlusion. The results also suggested that the proposed model could filter out uninformative features, since the recognition rate did not decline when input from Cam5 was combined with other views; using single-view input from Cam5 resulted in impaired performance of the proposed model.
